# 核糖体生物合成与靶向治疗在弥漫大B细胞淋巴瘤中的研究进展

**DOI:** 10.3760/cma.j.cn121090-20250827-00403

**Published:** 2026-04

**Authors:** 敏行 岳, 维莅 赵, 黎 王

**Affiliations:** 上海血液学研究所，组学与疾病国家重点实验室，转化医学国家重大科技基础设施（上海），上海交通大学医学院附属瑞金医院，上海 200025 Shanghai Institute of Hematology, State Key laboratory of Medical Genomics, National Research Center for Translational Medicine at Shanghai, Ruijin Hospital Affiliated to Shanghai Jiao Tong University School of Medicine, Shanghai 200025, China

## Abstract

核糖体生物合成是真核细胞蛋白质合成所必需的生物学过程，也是细胞内能量消耗较高的过程之一。近年来，多组学研究显示，弥漫大B细胞淋巴瘤（DLBCL）中核糖体生物合成相关通路普遍异常激活，且与c-Myc重排或高表达、mTOR信号持续活化及TP53通路异常等分子事件密切相关，同时也是化疗耐药和不良预后的影响因素。本文在概述核糖体结构、核糖体生物合成主要过程及其相关调控网络的基础上，重点综述核糖体生物合成异常在DLBCL发生发展中的作用，系统总结传统化疗药物（如铂类和蒽环类药物）、选择性RNA聚合酶Ⅰ抑制剂及XPO1抑制剂等干预策略在DLBCL中的临床前和临床研究进展，并讨论基于核糖体生物合成活性及TP53状态进行患者分层、联合治疗和耐药干预的潜在应用价值。现有研究提示，核糖体生物合成异常不仅参与DLBCL的发生发展，也可能成为疾病分层和治疗干预的重要依据，为DLBCL精准治疗相关研究提供参考。

## Abstract (en)

Ribosome biogenesis is an essential biological process for protein synthesis in eukaryotic cells and is also one of the most energy-consuming cellular processes. Recent multiomics studies have shown that ribosome biogenesis-related pathways are broadly upregulated in diffuse large B-cell lymphoma（DLBCL）. These alterations are closely associated with molecular events, such as c-Myc rearrangement or overexpression, persistent activation of mTOR signaling, and TP53 pathway abnormalities, and are correlated with chemoresistance and poor prognosis. This review summarizes the structure of ribosomes, the major steps of ribosome biogenesis, and the regulatory networks governing them, with a particular focus on the role of dysregulated ribosome biogenesis in the pathogenesis and progression of DLBCL. Additionally, we systematically review the preclinical and clinical evidence for therapeutic strategies targeting ribosome biogenesis in DLBCL, including conventional chemotherapeutic agents like platinum compounds and anthracyclines, selective RNA polymerase I inhibitors, and XPO1 inhibitors. The potential clinical value of patient stratification, combination therapy, and resistance interventions based on ribosome biogenesis activity and TP53 status is also discussed. Current evidence suggests that dysregulated ribosome biogenesis is involved in DLBCL development and progression and may serve as an important basis for disease stratification and therapeutic intervention, providing a reference for future studies on precision treatment in DLBCL.

近年研究揭示，核糖体生物合成通路在包括白血病、实体瘤及淋巴瘤在内的多种恶性肿瘤中普遍激活，且与患者不良预后显著相关[1]。弥漫大B细胞淋巴瘤（DLBCL）作为成人最常见的侵袭性B细胞淋巴瘤，约40％患者面临原发耐药或复发困境，传统的R-CHOP方案作为标准一线治疗虽有效，但在高危患者中疗效有限，嵌合抗原受体T（CAR-T）细胞疗法等新型治疗手段在复发难治性患者中展现出潜力[2]–[3]，然而其高昂成本及可及性障碍限制了临床广泛应用。有研究分析复发难治性DLBCL患者转录组信息显示核糖体生物合成通路处于激活状态，靶向抑制该过程被认为有助于克服传统化疗耐药；进一步分析通路机制，DLBCL细胞中核糖体生物合成的过度激活与癌基因（如c-Myc）重排、抑癌基因（如TP53）失活密切相关，并通过促进核糖体大量生成，从而驱动肿瘤细胞异常增殖和凋亡逃逸[4]–[6]。复发难治性DLBCL的转录组数据中核糖体生物合成通路基因（如RNA聚合酶Ⅰ复合物成员、核糖体RNA修饰酶）持续高表达，提示其可作为克服耐药的潜在靶点[1]。当前，靶向核糖体生物合成的治疗策略涵盖传统化疗药物（如铂类、蒽环类）及新型选择性抑制剂（如CX-5461）。这些药物通过干扰核糖体DNA转录、阻断核糖体组装或诱导核仁应激，在临床前模型中展现出显著抗DLBCL作用。然而，核糖体生物合成在DLBCL中的特异性调控网络及其与肿瘤微环境的互作仍待系统解析。本文聚焦核糖体生物合成在DLBCL中的致病机制，综述关键靶点药物研发进展，旨在为精准干预核糖体生物合成通路、改善DLBCL个体化治疗提供理论依据。

一、核糖体的合成过程及调控机制

核糖体由核糖体RNA（rRNA）与核糖体蛋白（RP）组成，其生物合成过程涉及前体rRNA加工修饰、核糖体蛋白合成及核内装配、核输出和亚基成熟等有序步骤，空间上跨越核仁、细胞核与细胞质[7]。在核仁内，RNA聚合酶Ⅰ转录核糖体DNA（rDNA）产生47S前体rRNA，经剪切生成18S、5.8S和28S rRNA；RNA聚合酶Ⅲ转录5S rRNA，RNA聚合酶Ⅱ则合成核糖体蛋白以及多种组装和修饰因子，以上装配成40S和60S前体亚基，并最终输出至细胞质形成功能性80S核糖体[7]–[8]。值得注意的是，多组学分析显示，参与rRNA加工和修饰的部分基因（如RMRP、fibrillarin等）在DLBCL中表达显著升高，提示DLBCL对高通量核糖体生成具有内在依赖性[1],[9]。

核糖体生物合成由TP53、c-Myc和mTOR三大关键因子构成的核心调控网络进行调控：c‑Myc通过同时增强RNA聚合酶Ⅰ/Ⅱ/Ⅲ转录，从而增加rRNA、核糖体蛋白和多种装配因子的表达；哺乳动物雷帕霉素靶蛋白复合物1（mTORC1）通过接收营养和生长因子信号，协调5′TOP mRNA翻译和rRNA合成；TP53及其上游的核糖体应激监控通路则在核糖体生成受阻时通过RPL5/L11-鼠双微体同源基因2（MDM2）-TP53轴触发细胞周期阻滞或凋亡，维持核糖体质量平衡[4],[10]–[11]。

二、核糖体生物合成与DLBCL的关系

1. 核糖体生物合成异常是肿瘤的共性特征：核糖体生物合成是细胞增殖的核心限速步骤，其异常激活已被视为肿瘤的“新标志”（Hallmark）[1]。泛癌研究表明，与正常组织相比，多数恶性肿瘤存在不同程度的核糖体生物合成增强，且该特征与较差的临床结局显著相关，在DLBCL等侵袭性B细胞淋巴瘤中尤为明显[10]。现有证据提示，TP53、c-Myc及mTOR等关键信号网络异常是肿瘤细胞核糖体生物合成持续活跃的重要分子基础[7],[10]。在乳腺癌、结直肠癌等实体瘤以及急性白血病、骨髓增生异常综合征等血液系统恶性肿瘤中，rDNA转录增强、核仁增大及rRNA修饰异常均与肿瘤进展、复发风险增加及预后不良密切相关[7],[12]–[13]。DLBCL中同样观察到类似的核仁形态学及分子改变，提示核糖体生物合成异常可能是其发生发展过程中的共性生物学基础之一[1]。

2. DLBCL中核糖体生物合成的独特调控机制：在DLBCL的发生发展中，c-Myc发挥了核心作用，5％～15％的DLBCL患者存在c-Myc基因重排或c-Myc蛋白高表达[14]，c‑Myc通过同时上调RNA聚合酶Ⅰ/Ⅱ/Ⅲ驱动rRNA、核糖体蛋白及多种装配因子（如出核蛋白XPO1）的转录，使DLBCL细胞维持持续的高通量蛋白质合成[1],[4],[12],[15]。此外，磷脂酰肌醇3-激酶（PI3K）-蛋白激酶B（AKT）-mTOR通路在部分DLBCL，尤其是活化B细胞样亚型（ABC型）及部分生发中心B细胞样亚型（GCB型）中持续活化，可进一步增强rDNA转录及核糖体蛋白相关mRNA的翻译效率，提示该通路参与DLBCL核糖体生物合成异常的维持[7],[16]。在此基础上，TP53状态与DLBCL细胞对核糖体生物合成抑制的反应密切相关。动物模型和临床研究表明，TP53野生型且核糖体生物合成水平较高的淋巴瘤细胞，在经过抑制rRNA合成或破坏核仁结构的药物（如蒽环类、多种铂类及选择性RNA聚合酶Ⅰ抑制剂CX-5461）处理后，更容易激活RPL5/RPL11-MDM2-TP53相关应答，并诱导细胞凋亡[4]。然而，DLBCL中TP53突变约占20％，相关异常可导致TP53功能受损[17]；因此，当核糖体生物合成过度激活时，如果TP53功能发生异常则无法通过RPL5/RPL11-HDM2轴有效介导凋亡程序，进而有利于肿瘤细胞存活[18]。已有研究提示，TP53失活还可能削弱核糖体生物合成抑制剂的促凋亡作用，使细胞反应更多表现为细胞周期阻滞[13]。

此外，DLBCL中还存在与核糖体生物合成密切相关的辅助调控。例如，XPO1在部分DLBCL中发生扩增或高表达，负责将多种rRNA加工因子、核糖体亚基及DNA损伤应答相关蛋白转运至细胞质，帮助肿瘤细胞在高复制压力和基因毒性治疗下维持核糖体稳态和基因组稳定；临床前研究显示，XPO1抑制剂与蒽环类、依托泊苷等化疗药物联合时，可显著增强DLBCL细胞的DNA损伤残留和凋亡，XPO1可能是连接核糖体生物合成异常与治疗反应的重要干预靶点[19]–[21]。综上，DLBCL中核糖体生物合成异常主要与c-Myc和PI3K-AKT-mTOR通路激活、TP53功能状态以及XPO1介导的适应性调控相关，这些分子共同构成了靶向核糖体生物合成治疗策略的重要生物学基础[5],[22]–[23]。

三、靶向核糖体生物合成的DLBCL治疗方案探究

目前已知可选择性或非选择性抑制核糖体生物合成的药物，其作用机制主要集中于抑制rDNA转录。根据DLBCL目前治疗情况将相关药物分为两类（表1）：一类是已经嵌入R‑CHOP、R‑CHOEP、R‑EPOCH以及基于铂类的挽救方案中的经典化疗药物，其核糖体靶向作用在近年的基础与临床研究中被重新认识；另一类则是以RNA聚合酶Ⅰ抑制剂、G‑四链体（G4）稳定剂和XPO1抑制剂为代表的新型小分子药物，目前主要处于DLBCL相关的临床前或早期临床探索阶段[4]。基于前文关于DLBCL核糖体生物合成异常及其调控网络的认识，理解这些药物的核糖体靶点有助于解释不同分子亚型DLBCL对治疗的差异性敏感性，并为后续的患者分层和联合用药策略提供理论依据。

**表1:** 靶向核糖体生物合成的治疗药物

药物种类	药物名称	作用机制	对RNA聚合酶Ⅰ介导转录的抑制机制	临床已有应用（是/否）
烷化剂	顺铂	DNA损伤及合成抑制	抑制UBF与rDNA结合	是
	奥沙利铂	DNA损伤及合成抑制	抑制UBF与rDNA结合	是
抗代谢物	5-FU	抑制胸苷合成	抑制rRNA修饰	是
	甲氨蝶呤	抑制二氢叶酸还原酶	未完全探明	是
抗生素	放线菌素D	与DNA结合	阻断rDNA转录延伸	是
	阿霉素	TOP2抑制剂	抑制rDNA转录起始	是
	米托蒽醌	TOP2抑制剂	抑制rDNA转录起始	是
选择性RNA聚合酶Ⅰ抑制剂	CX-3543	解离核仁素与rDNA	抑制rDNA转录起始	否，Ⅱ期临床试验
	CX-5461	干扰SL-1与RNA聚合酶的相互作用	阻断rDNA转录延伸	否，Ⅱ期临床试验
XPO1抑制剂	塞利尼索	出核蛋白XPO-1抑制剂	–	是

**注** UBF：上游结合因子；rDNA：核糖体DNA；5-FU：5-氟尿嘧啶；TOP2：DNA拓扑异构酶2；SL-1：TATA-box结合蛋白相关因子复合物；XPO1：核输出蛋白1；–：无内容

1. 铂类药物：顺铂及其类似物奥沙利铂等铂类烷化剂广泛应用于挽救性方案中，如R‑DHAP、R‑ICE和R‑GDP方案等复发难治性DLBCL患者常用治疗方案[4]。铂类化疗药物也被广泛应用于多种死亡率高的癌症中，如小细胞肺癌、卵巢癌和头颈部鳞状细胞癌等[24]–[25]。铂类化合物通过形成铂-DNA加合物发挥作用，加合物被DNA损伤修复蛋白识别并结合，最终通过诱发细胞周期阻滞或抑制蛋白质合成等机制导致细胞死亡[26]。近期研究表明，铂类化合物，尤其是奥沙利铂，可通过抑制rRNA转录发挥抗肿瘤效应，即通过置换rDNA位点上的转录因子上游结合因子（UBF），抑制rDNA转录，并破坏rDNA处的染色质开放状态，进一步激活核仁应激通路（NSP），并介导下游的细胞周期阻滞及凋亡等反应[27]–[28]。

2. 抗代谢类药物：5-氟尿嘧啶（5-FU）、甲氨蝶呤（MTX）通过模拟天然代谢物结构，竞争性抑制特定代谢酶活性，从而干扰DNA合成。5-FU及其活性代谢物可被整合入RNA与DNA，或结合胸苷酸合成酶的活性位点抑制其功能，导致脱氧胸苷三磷酸（dTTP）合成减少，造成DNA合成所需的胸苷酸缺乏。上述机制共同导致RNA和DNA损伤，最终引发肿瘤细胞死亡[29]–[30]。因此，5-FU可通过在rRNA加工水平阻断47S rRNA的成熟来影响核糖体生物合成[31]。MTX的作用机制与5-FU不同，其主要通过抑制二氢叶酸还原酶，阻碍叶酸的还原利用。叶酸缺乏导致胸苷酸合成酶无法将脱氧尿苷酸转化为脱氧胸苷酸，同样导致DNA合成障碍。实验表明，MTX处理细胞可以降低RNA聚合酶Ⅰ介导的rDNA转录水平，并诱导核仁结构破坏，抑制核糖体生物合成[31]。在DLBCL的临床实践中，高剂量MTX联合阿糖胞苷（Ara‑C）的方案常用于中枢神经系统侵犯或高危DLBCL的挽救治疗，其部分作用机制可能依赖于对高负荷核糖体生物合成的多靶点打击，即阻断核苷酸供应的同时直接抑制RNA聚合酶Ⅰ转录并诱导核仁解体[4]。精准治疗视角下，对于具有高核糖体活性且肾功能稳定能耐受的年轻高危患者，适度加强含MTX/Ara‑C方案有望增强核糖体应激-TP53轴的抗肿瘤效应，而在TP53突变或伴显著骨髓储备不足的患者中，则需谨慎权衡不良反应与获益[7]。

3. 抗生素：DNA嵌入型抗生素包括蒽环类药物阿霉素以及放线菌素D等，被纳入多种淋巴瘤化疗方案中。在DLBCL的一线R‑CHOP方案中，阿霉素是重要的细胞毒性成分之一[4]。除经典的拓扑异构酶Ⅱ抑制和DNA互链交联作用外，这类药物还可富集于核仁，抑制rDNA转录并诱导核仁破坏，其中放线菌素D在低剂量时发挥选择性RNA聚合酶Ⅰ抑制作用，被广泛用于研究核糖体生物合成应激。Derenzini等[4]通过系统分析发现核糖体生物合成基线活性越高的血液肿瘤，对蒽环类和放线菌素D诱导的核糖体应激和p53激活越敏感，这与DLBCL在标准R‑CHOP方案治疗中高比例可治愈的临床观察相吻合。因此，将阿霉素等经典药物重新理解为“嵌入式核糖体生物合成抑制剂”，有助于解释部分DLBCL患者对一线方案高度敏感，而另一部分伴TP53缺陷或核糖体监控通路异常的患者则更易出现原发耐药或早期复发的情况[7]。

4. RNA聚合酶Ⅰ选择性抑制剂：随着对核糖体生物合成及RNA聚合酶Ⅰ介导的转录过程在肿瘤中普遍上调的认识加深，以及许多现有的化疗药物被证实具有显著的抗RNA聚合酶Ⅰ转录活性，选择性抑制RNA聚合酶Ⅰ的药物被认为有望成为一类新的抗癌疗法，其疗效可能优于非选择性抑制剂且不良反应更小。目前进入临床试验的RNA聚合酶Ⅰ选择性抑制剂主要有CX-3543和CX-5461。CX-3543能够将核仁素从rDNA位点的G4结构上解离，从而特异性抑制rDNA转录[32]。G4是由富含连续鸟嘌呤（G）的DNA或RNA单链折叠形成的四链螺旋结构，可影响包括转录在内的多种细胞过程。例如，c-Myc基因启动子区的G4结构可抑制转录起始，而rDNA基因上的G4结构则会促进rRNA的转录[33]。CX-3543在多种肿瘤细胞系中显示出显著的生长抑制活性，并在体内模型实验中被验证具有抗肿瘤效果[34]。

CX-3543虽已成功完成Ⅰ期临床试验，并曾进入针对神经内分泌肿瘤和类癌的Ⅱ期临床试验（NCT00780663），但最终因生物利用度问题而撤出。CX-5461作用机制与CX-3543不同。实验证明，在低浓度下（IC_50_<100 nmol/L），CX-5461可有效抑制多种肿瘤细胞的RNA聚合酶Ⅰ转录活性，其对RNA聚合酶Ⅰ的选择性抑制效能是RNA聚合酶Ⅱ的200倍以上[27]。目前认为，CX-5461通过特异性干扰SL-1与RNA聚合酶Ⅰ的相互作用，从而抑制RNA聚合酶Ⅰ转录复合物形成及rDNA转录延伸。研究证明，在TP53野生型癌症中，CX-5461可通过诱导核仁应激，促使RPL5和RPL11降解人双微体基因2（HDM2），稳定TP53蛋白并激活下游细胞凋亡通路[13]。而在TP53缺失的癌症中，CX-5461则通过激活ATM/ATR介导的信号传导通路导致细胞周期阻滞[18]。CX-5461已在多种不同TP53状态的肿瘤细胞中显示出抗增殖的活性[34]。该药已完成针对晚期血液系统恶性肿瘤的临床试验，目前正在进行针对乳腺癌的Ⅰ/Ⅱ期临床试验（NCT02719977）[35]。

综上，DLBCL当前的治疗谱系同时涵盖了“嵌入式”与“专属性”两类核糖体靶向策略：一方面，阿霉素长期构成DLBCL一线免疫化疗方案的重要组成，而顺铂、MTX等药物则常用于部分挽救治疗或特定临床情境。研究表明，此类药物除经典的基因毒性作用外，还可通过抑制rDNA转录、破坏核仁稳态并激活核糖体应激-TP53通路来发挥抗肿瘤效应[28]；对于TP53野生型且核糖体生物合成较为活跃的DLBCL，这一作用可能与此类药物较好的治疗敏感性相关。另一方面，以CX-5461为代表的RNA聚合酶Ⅰ选择性抑制剂和XPO1抑制剂如塞利尼索在多发性骨髓瘤中被证明可以通过抑制核糖体生物合成抑制肿瘤增殖[22]。推动以核糖体生物合成为中心的新型分层与联合治疗，未来有望在高危或复发难治性DLBCL中实现更具机制导向的精准用药。

四、总结与展望

作为最常见的血液系统恶性疾病，DLBCL的核糖体生物合成活跃度在众多癌种中位居前列[1]，而c-Myc高表达、TP53突变等特征也进一步加剧了DLBCL的不良预后。值得关注的是，对于部分已用于临床的化疗药物（如阿霉素、铂类、塞利尼索等），其抑制肿瘤细胞核糖体生物合成的作用机制正在被积极揭示并已取得进展。同时，基于高通量的小分子筛选，也为开发新型选择性抑制RNA聚合酶Ⅰ提供了新的可能。鉴于核糖体生物合成过程涉及多个步骤（如rRNA修饰、RNA与RP结合、核糖体前体成熟），除抑制转录外，靶向其他环节同样具有治疗潜力，有望改善患者由于核糖体生物合成过度激活而导致的原发耐药和复发的情况，从而提升化疗获益，进一步达到个性化用药和精准治疗。

然而，靶向核糖体生物合成成功转化为临床实践仍面临诸多挑战。药物选择性上，虽然CX-5461等新型抑制剂对RNA聚合酶Ⅰ展现出高选择性，但增殖活跃的正常细胞（如造血干细胞、胃肠道黏膜细胞）同样依赖高效的核糖体生物合成，因此如何平衡抗肿瘤活性与全身不良反应是实现临床应用的关键[34]。其次，耐药性不容忽视。肿瘤细胞可能通过激活旁路信号（如增强mTORC1活性）、突变RPL5/RPL11-HDM2轴或强化DNA损伤修复能力来逃逸RNA聚合酶Ⅰ抑制诱导的细胞死亡[13],[36]。目前仍缺乏可靠的生物标志物来精准预测疗效。尽管c-Myc过表达和野生型TP53被普遍认为是敏感指标，但考虑到DLBCL患者群体的异质性，上述指标并不足以实现完美的患者分层。此外，核糖体生物合成与肿瘤微环境（如免疫细胞浸润）间的相互作用尚不明确，抑制肿瘤细胞的核糖体合成是否会影响其抗原呈递能力，进而改变免疫治疗的疗效，仍是未知并需极力探索的方向。

总体而言，针对核糖体生物合成的DLBCL治疗目前大致处于3个发展阶段：其一是“重新解读”传统方案，将顺铂、蒽环类等经典药物的核糖体靶向效应纳入DLBCL精准治疗框架；其二是通过CX‑5461等选择性RNA聚合酶Ⅰ抑制剂、塞利尼索等XPO1抑制剂，在复发难治性及高危DLBCL中开展机制驱动的早期临床研究；其三是面向未来，结合多组学与功能筛选，挖掘更精细的核糖体生物合成调控节点，并探索与免疫检查点抑制剂、CAR‑T细胞治疗等免疫疗法的联合，使“靶向核糖体”真正从基础概念走向可操作的分层治疗策略。
